# From seeing to believing: labelling strategies for *in vivo* cell-tracking experiments

**DOI:** 10.1098/rsfs.2013.0001

**Published:** 2013-06-06

**Authors:** Fränze Progatzky, Margaret J. Dallman, Cristina Lo Celso

**Affiliations:** Department of Life Sciences, Imperial College London, London SW7 2AZ, UK

**Keywords:** cell biology, *in vivo* imaging, cancer, stem cells, fluorescence microscopy

## Abstract

Intravital microscopy has become increasingly popular over the past few decades because it provides high-resolution and real-time information about complex biological processes. Technological advances that allow deeper penetration in live tissues, such as the development of confocal and two-photon microscopy, together with the generation of ever-new fluorophores that facilitate bright labelling of cells and tissue components have made imaging of vertebrate model organisms efficient and highly informative. Genetic manipulation leading to expression of fluorescent proteins is undoubtedly the labelling method of choice and has been used to visualize several cell types *in vivo*. This approach, however, can be technically challenging and time consuming. Over the years, several dyes have been developed to allow rapid, effective and bright *ex vivo* labelling of cells for subsequent transplantation and imaging. Here, we review and discuss the advantages and limitations of a number of strategies commonly used to label and track cells at high resolution *in vivo* in mouse and zebrafish, using fluorescence microscopy. While the quest for the perfect label is far from achieved, current reagents are valuable tools enabling the progress of biological discovery, so long as they are selected and used appropriately.

## 1. Introduction

The long-standing enthusiasm for *in vivo* microscopy (IVM) results from the unique perspective that can be gained when observing biological phenomena evolve in real time under physiological conditions. Simple bright field illumination *in vivo* imaging was first reported in 1839 [1]. The same approach was described for studying leucocytes rolling along blood vessel walls in 1972 [2]. In the following decades, the advent of fluorescence microscopy and the discovery and development of multiple fluorophores made IVM a more versatile experimental methodology [3]. For instance, the development of confocal microscopy improved contrast and optical resolution of microscope images by reducing the out-of-focus signal. Two-photon microscopy was developed to increase penetration depth, and, in addition, allowed detection of collagen fibres in the extracellular matrix through second-harmonic generation (for details about confocal and two-photon fluorescence microscopy, see [4]). Single-cell resolution IVM has been widely used to understand immune responses, tissue architecture and turnover, tumour development and stem cell behaviour. Hence, IVM is an invaluable tool to study complex biological processes involving the interaction of multiple cell types and to assess the efficacy of novel therapeutic protocols.

The two vertebrate model organisms currently most widely used for IVM studies are mouse and zebrafish; the mouse primarily for its similarity to humans and the zebrafish for its small size, *ex utero* development, transparent embryos and the availability of transparent adult mutants. Transgenic mouse and zebrafish reporter lines expressing fluorescent proteins in cell lineages of interest are ideal tools for IVM experiments. However, because the generation of transgenic animals is costly and time-consuming, experimental models based on syngeneic or xenotransplantation of cells are often favoured. Cells stably expressing fluorescent proteins can be transplanted into either wild-type or fluorescent reporter recipient animals, which markedly expand the number and types of cells that can be monitored simultaneously. Moreover, xenotransplantation is the only available experimental system for tracking human cells in these experimental animals, and several generations of immunocompromised and humanized genetically modified mice have been created to improve human cell engraftment [5–7]. In the past decades, the use of zebrafish as recipient organism has gained increasing popularity because of its amenability to experimental procedures for high-throughput screening purposes [8]. Optical translucency, *ex utero* development and the small size of zebrafish larvae allow imaging of cell engraftment, proliferation and migration in real time at the single-cell level, *in vivo* in the intact organism. In addition, the availability of transparent mutant and transgenic zebrafish lines readily allows investigation of the interaction of host and transplant cells, not only at the embryonic stage but also into adulthood [9,10].

The now widespread use of fluorescence-based IVM results from an ever-growing array of available fluorophores that can be used to label cells and tissue structures. These fluorophores can roughly be divided into two categories: endogenous reporters, i.e. fluorescent proteins constitutively produced by the cells of interest; and exogenous probes, i.e. chemicals that interact with cellular or tissue components (the latter being normally applied during *ex vivo* reactions followed by injection of labelled cells into a recipient organism). Independent of their type and specific use, all IVM fluorophores need to be non-toxic, photostable (i.e. resistant to photobleaching) and sufficiently bright to generate a signal detectable through living tissues. In the following, we review fluorescent proteins and dyes successfully reported to allow *in vivo* imaging at single-cell resolution and discuss promising developments that are likely to further improve the field.

## 2. Fluorescent protein-based reporters

### 2.1. Green fluorescent protein reporters

The green fluorescent protein (GFP) from the jellyfish *Aequorea victoria* [11], especially in its stabilized and enhanced variants, is the most widely used fluorescent reporter in biological research. Transgenic mice and zebrafish expressing GFP in specific cell lineages or whole tissues have been extensively used for IVM experiments over the past few decades. For example, MMTV-GFP mice were used to visualize mammary gland cells and tumours [12], and GFP-M mice to visualize neurons within the central nervous system [13]. Vasculature has been highlighted in mice with the Tie2-GFP transgene [14] and in zebrafish with the fli1:EGFP transgene [10]. Labelling of myeloid and lymphoid cells has been critical in following their behaviour *in vivo* and in understanding how organisms respond to infections. For instance, Lys-GFP and CSF-1R-GFP mice have allowed single-cell resolution of myeloid cells and macrophages, respectively [14], and CD2-GFP and FoxP3-GFP mice of inflammatory and regulatory T lymphocytes [15,16]. In zebrafish, mpx:GFP and lyz:GFP transgenic lines have been generated to identify predominantly neutrophils [17,18], and the mpeg1:EGFP transgenic lines to track macrophages [19]. Ubi:EFGP zebrafish and ubi-GFP mice allow cell tracing following adoptive transfer and Cre-loxP lines combined with flox-STOP-flox-eGFP or YFP lines greatly increased the breadth of lineage-tracing studies [20,21].

**Figure 1.**
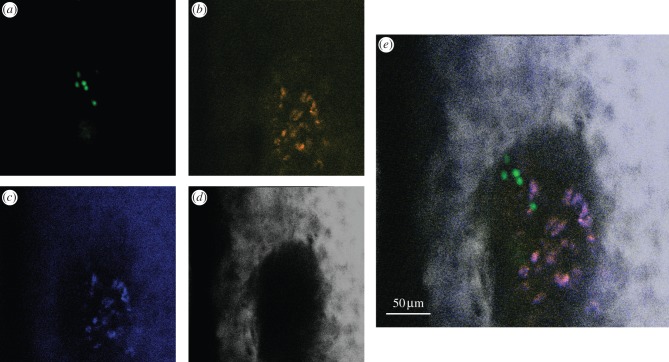
Transgenic expression of a fluorescent protein allows *in vivo* imaging of cells several days following transplantation. Rosa26 rtTA TetO H2BGFP long-term repopulating haematopoietic stem cells (LT-HSCs) expressing doxycycline-inducible H2BGFP were purified by fluorescence-activated cell sorting, labelled with DiD and injected into a wild-type, doxycycline-fed lethally irradiated recipient mouse (see [23] for experimental details). Eight days following transplantation, engrafting clones were detectable through bright (*a*) GFP nuclear signals, whereas DiD is diluted to undetectable levels, and (*b*) the signal collected in the DiD channel is generated by autofluorescent cells (autofluorescence signal (*c*) completely overlaps the signal collected in the DiD channel). (*d*) Second-harmonic generation signal originates from bone collagen and is detected to identify the edges of the bone marrow cavity. (*e*) Merge of all four channels.

Restriction of the GFP signal to the cell nucleus is particularly useful for tracking cells that are tightly packed together and to concentrate the fluorescent signal in a smaller, brighter area, making it more easily detectable in deep tissues. Nuclear expression of GFP can be achieved with a variety of strategies, the most widely used being the fusion protein histone 2B-GFP (H2BGFP). Rompolas *et al.* [22] took advantage of constitutive H2BGFP expression as a nuclear tracer to monitor orientation of cell division and daughter cell localization in the hair follicle bulge and body throughout its cycle. H2BGFP provides a sufficiently strong signal to allow detection of the progeny of engrafting haematopoietic stem and progenitor cells (HSPCs) within mouse bone marrow, at times when a dye-based label is diluted below detectable levels (figure 1). Recently, it has become possible to monitor not only single cells but also subcellular structures and processes, for example to detect exocytosis (ubiquitous and Glut4-eGFP transgenic mice [24,25]) and autophagy (LC3-GFP transgene in zebrafish and mice [26,27]).

GFP has been used not only to mark specific cell types within living tissues, but also to monitor signalling events. TOP:dGFP transgenic zebrafish carry a destabilized GFP coding sequence downstream of an artificial promoter containing seven repeats of a TCF/LEF binding sequence, activated in response to Wnt signalling. These fish have been used to monitor waves of Wnt signalling during embryonic development [28]. Owing to its signal strength, the H2BGFP fusion protein has recently been used to visualize Wnt signalling events within living TCF/LEF:H2BGFP mice [29]. This mouse reporter system provides a major advance because previous Wnt pathway markers were detectable only in histological sections or in cultured cells [30,31]. However, it may be temporally less accurate, because the H2BGFP protein has a long half-life [32], which could lead to the fluorescent signal persisting for longer than the Wnt signal itself.

### 2.2. Multi-colour labelling strategies

The generation of blue/yellow-shifted GFP variants (blue, cyan and yellow fluorescent proteins), together with the discovery- and mutation-based development of red fluorescent proteins isolated from tropical corals and anemones, has expanded the palette of colours available and has allowed the development of more complex reporter strategies (table 1).

**Table 1.** Examples of fluorescent proteins and dyes used for single-cell resolution IVM experiments. The excitation and emission peaks are indicated, together with references to some examples taken from available literature. For photoswitchable/photoconvertible proteins, excitation and emission peaks both before and after conversion are indicated.

	excitation peak (nm)	emission peak (nm)	*in vivo* imaging	
			mouse	zebrafish
*fluorescent proteins*				
Cerulean	433	475		[33]
GFP (EGFP)	396 (488)	508	[12–14,34]	[10,17–19,28,35]
Emerald	487	509	[36]	[37]
Azami-Green	492	505	[38]	
YFP	514	527	[39]	[40]
Venus	515	528	[41]	[42]
mKO	548	559		[43]
Kusabira-Orange	548	561	[38]	
mOrange1	548	562		[44]
tdTomato	554	581	[45]	[46]
Katushka	558	635	[47]	
dsRed2	563	582	[48]	[18]
mRFP1	584	607	[34,49]	
mCherry	587	610	[34,49]	[50,51]
mKate	588	635		[52]
mPlum	590	649	[34]	
Neptune	600	650	[53]	
eqFP650/670	592/605	650/670	[54]	
*photoswitchable fluorescent proteins*				
	before/after photoconversion			
PS-CFP2	400/490	468/511	[55] (chicken)	
Dendra2	490/553	507/573	[56]	[33,57]
Dronpa	dark/503	dark/518		[58]
PA-GFP	dark/504	dark/517	[59]	
Eos	506/571	516/581		[60]
Kaede	508/572	518/580	[61]	[62]
KikGR	507/583	517/593		[63]
PSmOrange	548/634	565/662	[64]	
*dyes*				
Qdots/nanoparticles	multiple	multiple	[65]	
BODIPY	multiple	multiple	[66]	[67,68]
Hoechst 3342	350	461	[69]	
DiD	456	591		
DiO	484	501		[70]
CFSE	494	515	[71,72]	
CellTracker Orange	548	576	[71]	
DiI	549	565	[36]	[73]
PKH26	551	567	[74]	
CM-DiI	553	570		[70,75]
DiR	748	780	[76]	

Apart from simultaneous visualization of multiple cell types [19,20,50], the extended palette of fluorescent proteins available has led to some very elegant applications from labelling different phases of the cell cycle to different clones of cells within a tissue. For instance, the FUCCI reporter system, which is based on the combination of two fusion proteins, containing the ubiquitination domains of human Geminin and Cdt1 fused to Azami-Green (AG) and Kusabira-Orange (KO) fluorescent proteins, respectively, allows real-time visualization of cell cycle progression in living cells [38]. A human tumourigenic cell line stably expressing the FUCCI reporter system was used to monitor the proliferative status of tumours implanted subcutaneously in mice [77]. While the first generation of the FUCCI reporter system is still primarily being used to study cultured cells, FUCCI transgenic zebrafish have allowed the study of proliferation waves in early developmental stages [43]. Recently, a new type of FUCCI reporter strategy, based on the use of mCherry and Venus fluorescent proteins, has allowed similar studies to be performed within live, cultured mouse embryos [78]. Application of the FUCCI technology to the study of living adult tissues will allow understanding of the fine details of tissue dynamics, from stem and progenitor cell proliferation patterns to tumour progression and response to treatment.

By combining multiple fluorescent proteins, from blue to far red, there are virtually no limitations to the number of colours that can be used to mark specific cell types, and it is now possible not only to identify cells belonging to different lineages or transitioning through different cell cycle stages, but also to uniquely label and monitor multiple clones within the same tissue. Lentiviruses encoding Cerulean, eGFP, Venus, tdTomato and mCherry have been used to concurrently transduce haematopoietic cells *ex vivo*, with different clones expressing high levels of unique combinations of the fluorophores. Such cells subsequently transplanted into lethally irradiated recipient mice can be monitored using both *in vivo* and *ex vivo* approaches [79]. Such work has indicated that single clones of engrafted haematopoietic cells can occupy large bone marrow regions. Interestingly, even though cells carrying multiple fluorescent proteins were transplanted, clones carrying a single fluorescent protein tended to be dominant, suggesting that overexpression of multiple fluorescent proteins may confer a disadvantage when clones compete for engraftment.

Brainbow and confetti transgenic mice and zebrafish carry tandem repeats of multiple fluorescent proteins (typically blue, green, yellow and red), which are combinatorially expressed following cre-mediated recombination [80–82]. Histological studies have shown efficient recombination and unique expression patterns in adjacent clones, indicating low toxicity of this system. Provided the levels of expression of all fluorescent proteins are sufficient for *in vivo* detection, these transgenic animals are promising candidates for future studies aimed at assessing physiological tissue dynamics.

### 2.3. Forster resonance energy-transfer-based and photoactivatable reporters

One of the main advantages of IVM studies is the possibility of monitoring biological processes as they happen. In this respect, imaging selected signalling events in real time and marking specific cells of interest to track their behaviour have been some of the most sought-after developments in the field. Cell signalling events have been documented in IVM studies by combining pairs of fluorescent proteins able to undergo Forster resonance energy transfer (FRET). Within a FRET pair, the excited-state energy of the blue-shifted (donor) protein is transferred to the red-shifted (acceptor) protein, leading to a unique emission pattern. Energy transfer is possible only if the two proteins are adjacent to each other, and therefore FRET has long been used for co-localization as well as for protein activation studies. *In vivo*, it has been used to visualize signalling events, protein–protein interactions and protein activation. For example, CFP-YFP and GFP-RFP FRET sensors have been used to study Rho GTPase activation in zebrafish embryos and in mouse tumours, respectively [83,84]. Caspase and calpain proteolytic activities in mice were investigated, using FRET sensors based on eGFP–tHcred1 and eCFP–eYFP donor–acceptor pairs, respectively [85,86].

Photoswitchable or photoconvertible fluorescent proteins allow the labelling of specific cells so that they can be identified through multiple imaging sessions. Such proteins change their fluorescence properties when excited at specific wavelengths (usually ultraviolet) changing from a ‘dark’ to a fluorescent state or from one fluorescent excitation/emission spectrum to another. For example, Dendra2 expressing tumour cells were used to study migratory patterns within mammary tumours [56], transgenic mice expressing photoactivatable GFP (PA-GFP) were used to track B cells migrating between different areas of lymph node germinal centres during maturation [59]. Photoactivatable Kaede was used to label cells residing in specific lymph nodes or skin areas in mice so that they could be recognized following migration to other lymphoid organs using flow cytometry [61]. In zebrafish, Dronpa was used to label individual neurons allowing reconstruction of the fish neuronal network [58] and Dendra2 to visualize the origin and fate of neutrophils during induction and resolution of inflammation [57]. H2B-Dendra2 transgenic zebrafish were generated to monitor the migration and proliferation of tail fin cells contributing to tissue regeneration following injury [33].

## 3. Gene-transfer-free cell-labelling approaches

While an invaluable tool for elegant, long-term cell-tracking experiments that monitor cell behaviour under physiological conditions, the use of fluorescent proteins to label cells for *in vivo* tracking has a number of limitations. Transgenic organisms carrying fluorescent reporters for the cells of interest are ideal, but not all currently available reporters give a sufficiently strong signal to allow efficient detection *in vivo*. Stable expression of fluorescent proteins in cells of interest can be obtained through *ex vivo* manipulation (transfection/transduction, followed by population or clonal selection) and has been used by groups working with well-established cell lines [87]. However, especially in the case of primary cells such as haematopoietic stem cells (HSCs) or human cancer cells, laborious *in vitro* engineering is not always possible. Gene-transfer-free cell-labelling approaches have therefore been sought to obtain rapid, efficient and uniform *ex vivo* labelling of cells prior to transplantation and *in vivo* imaging. They provide the labelling of choice for cells that are hard to culture, and they also facilitate experimental approaches based on high-throughput screening of transplanted cells [88,89].

Dyes present a variety of different challenges and limitations in comparison with endogenously expressed fluorescent proteins, ranging from higher cytotoxicity to rapid dilution upon cell proliferation. However, they are often chosen because of their ease of use and the wide array of colours they provide. Even though many fluorescent dyes have been widely used for *in vivo* experiments based on flow cytometry readouts, they are not all suitable for IVM experiments because of the different fluorescent properties required for the two techniques. Fluorophores that photobleach can be used for flow cytometry because they only need to be excited and emit a signal once and briefly. By contrast, IVM fluorophores need to be photostable for high quality images to be collected (relatively) slowly, often through averaging signals, and to allow prolonged monitoring of the same cell over time.

### 3.1. Dye dilution and toxicity

**Figure 2.**
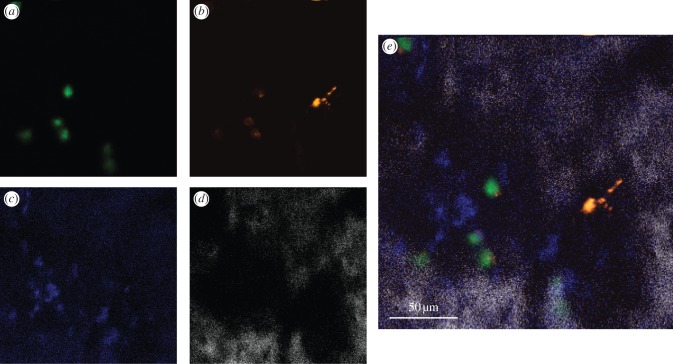
Non-specific DiD signal can be identified based on its shape. Rosa26 rtTA TetO H2BGFP LT-HSCs were labelled with DiD, injected into a wild-type, doxycycline-fed, lethally irradiated recipient mouse and imaged 5 days later. (*a*) GFP, (*b*) DiD, (*c*) autofluorescence and (*d*) SHG signals were collected. Bright, irregularly shaped DiD signal is not autofluorescence and represents cell debris. Live engrafting cells are clearly recognizable through GFP nuclear labelling and correspond to round-shaped DiD signal, of consistent size. (*e*) Merge of all four channels.

Dilution of dyes upon cell division is widely exploited *in vitro* and *in vivo* to study cell proliferation, because halving of cellular fluorescence following each cell division can be easily observed and quantified using flow cytometry [90]. Such an approach is unsuitable for IVM studies (figure 2) because the fluorescent signal obtained from cells within a tissue depends not only on their intrinsic brightness, but also on the nature of the surrounding tissue. This can lead to unpredictable signal loss owing to scattering and absorption by autofluorescent components. Single-cell resolution IVM can provide an indication of the proliferative history of labelled cells only if all cells belonging to each clone cluster together and the resulting clones remain separate from each other. Alternatively, flow cytometry can be used to assess dye dilution at the population level on recovered cells after *in vivo* imaging is completed.

While dye dilution is unavoidable, much can be done with respect to the toxicity of cell-labelling dyes. For example, dyes may affect different cellular functions and therefore dyes not affecting the function under study should be preferred. Preliminary dye titration experiments can identify a concentration window in which the staining is sufficiently strong, but toxicity is minimized. The chemical compounds used can be subdivided based on the cell compartment they label: cytoplasm, nucleus and cell membrane, each class with its own specific advantages and disadvantages.

### 3.2. Cytoplasmic and nuclear dyes

Cytoplasmic and nuclear dyes are, in principle, ideal for *in vivo* single-cell tracking because they should provide the most uniform staining; however, they tend to present higher cytotoxicity and are rarely used for intravital confocal and two-photon microscopy. Carboxyfluorescein succinimidyl ester (CFSE) and carboxyfluorescein diacetate succinimidyl ester (CFDA-SE) are amine-reactive probes that penetrate cells, where they are metabolized to amine-reactive chemicals, which then covalently bind to cytosolic components. High concentrations of CFSE have been associated with severe toxicity [91]; however, once carefully optimized, CFSE labelling enables *in vivo* cell tracking over a long period of time because it is very efficiently retained within the cytoplasm [92]. CFSE and CFDA-SE have been frequently used for flow cytometry-based proliferation studies because they are consistently halved between daughter cells giving rise to easily quantifiable peaks of decreasing intensity as more cell divisions occur [90]. Both CFSE and CFDA-SE are easily photobleached and their fluorescence decays vary rapidly with tissue depth *in vivo* because of the overlap of their excitation and emission spectrum with that of autofluorescent tissue components [93]. For this reason, they have been used for IVM protocols only in short-term studies of HSPCs observed through thinned bone or via bone marrow endoscopy [71,72].

Amine-reactive dyes, available with a wide spectral range and in far red-shifted variants, overcome the limitations of CFSE/CFDA-SE. Even though they are not excitable by two-photon wavelengths, they have been successfully used for confocal IVM experiments. For example, labelling and *in vivo* imaging of the intracellular pathogen *Listeria monocytogenes* in mouse spleens has been performed using BODIPY-630 [66].

BODIPY fluorophores conjugated to a cholesteryl ester and a sphingolipid (ceramide) have been used to visualize lipid accumulation in zebrafish gut [67] and visualize the zebrafish retina *in vivo* [68], respectively. As an alternative for imaging zebrafish retinal cells *in vivo*, fluorescent coumarin derivates have been successfully applied [94]. Cell tracker dyes are thiol-reactive and exist in blue, green and red variants. Their manufacturer indicates that labelled cells should be viable for ‘at least’ 24 h and, accordingly, CellTracker Orange has been successfully used for short-term imaging of HSPCs in mouse bone marrow and for imaging B cells in tumour-associated lymph nodes [69,71].

Dyes with high affinity for double-stranded DNA provide a bright nuclear signal, and therefore should be ideal for tracking cells within densely clustered populations and in deep tissues, but have been consistently reported to have a number of limitations when applied to *in vivo* single-cell resolution imaging. In fact, DNA-binding dyes can interfere with fundamental biological processes, including DNA replication and transcription, and can induce DNA damage. For example, Hoechst 33342 has been reported to inhibit the proliferation of mammalian cells at high concentrations [92] and DRAQ5 was shown to interfere with DNA-binding proteins such as histones, DNA repair, replication and transcription factors as well as essential cellular enzymes resulting in the inhibition of cellular functions [95,96]. Hoechst 33342 has the advantage of absorbing light in the UV range, excitable by two-photon microscopy, and is resistant to quenching. For these reasons, it has therefore been widely used to study lymphocyte migration, a process uncoupled from DNA dynamics and therefore not affected by nuclear dyes [69]. Moreover, Hoechst 33342 has been successfully used to visualize apoptosis *in vivo* [69].

### 3.3. Membrane dyes

Lipophilic, carbocyanine fluorescent tracking dyes, whose aliphatic portion binds to the cell membrane lipid bilayer, have been widely used by the scientific community and seem to have lower cytotoxicity than cytoplasmic and nuclear dyes [97]. PKH lipophilic dyes such as PKH2, PKH67 (green), PKH3 and PKH26 (red) have been extensively used for tracking lymphocytes [92]. PKH26 has been successfully used to visualize *in vivo* homing and proliferation of HSPCs [74]. These dyes stain the whole plasma membrane of cells through lateral diffusion, and also spread to intracellular organelles as a consequence of membrane turnover. Like CFSE, membrane dyes can be used as proliferation markers because, after each cell division, the fluorescence of daughter cells should be halved compared with that of the mother cell [98]. However, in our hands, the dilution peaks generated with membrane dyes are not as clearly defined, probably because of imperfect subdivision, local membrane dynamics and potential shedding or because of differential metabolism in daughter cells. For example, PKH26 has been shown to label cells non-uniformly [92] and this could account for uneven distribution between daughter cells.

More recently, another series of carbocyanine lipophilic dyes, DiO, DiI, DiD and DiR (green, red, far red and near-infrared emission, respectively), have become increasingly popular and are now routinely used for transplantation and *in vivo* tracking and imaging studies in mouse and zebrafish [36,75,99–101]. They all follow the same labelling principle and cover a large spectral range, thus facilitating tracking of multiple cell populations simultaneously through multi-colour imaging. Some of these dyes, for example DiR, are highly photostable, and all are easy and rapid to apply. Most importantly, despite the fact that they heavily intercalate within the lipid bilayer of cell membranes, they have not been reported to cause serious cytotoxicity at concentrations that provide strong, uniform staining. Because both the toxic and optimal labelling concentrations may vary from cell type to cell type, it is desirable to perform initial titration studies that identify the optimal working concentration for the cells of interest [27]. We and others have used them extensively to label HSPCs, and to track them within the bone marrow of live mice [36,101–105] or within peripheral blood vessels [106]. CM-DiI, a form of DiI resistant to fixation, has become particularly useful for staining xenografted tumour cells in zebrafish. Haldi *et al.* [75] were first to label human melanoma cells with CM-DiI and detect them *in vivo* up to day 4 post-injection, after which cells were visualized by double staining, using a human melanoma-specific anti-chondroitin sulphate antibody. Metastatic prostate cancer [107,108], pancreatic cancer [109], ovarian carcinoma [110,111], leukaemia [112,113] and breast cancer [108,111] cell lines have all been stained with CM-DiI, using a variety of protocols, and successfully transplanted into and imaged within zebrafish embryos.

## 4. Experimental controls for transplantation experiments based on lipophilic dye labelling in mouse and zebrafish

The use of cyanine lipophilic dyes for *ex vivo* labelling of cells for transplantation and IVM studies has become very popular owing to the easy and rapid staining procedures required and the ease of dye detection. Before selecting these dyes for routine use, however, it is important to consider a few points that may affect the results obtained.

### 4.1. Dye transfer and non-specific staining

One of the main considerations when using fluorescent chemicals to label cells is that the dyes can be released into the tissue, either the extracellular space or phagocytic vesicles inside other cells. Because dyes tend to be relatively resistant to degradation and may not lose their fluorescence once they are in a different environment, this can lead to a non-cell-associated signal, whose identification is critical for the generation of sound data. That transfer between cells can occur both *in vivo* and *in vitro* has been described especially in the case of lipophilic membrane dyes, both PKH and Di dyes [114,115]. This is not surprising, given that direct contact of cell membranes occurs between adjacent cells and can lead to the exchange of lipid components. Examples of membrane exchange have been reported for haematopoietic cells cultured on stroma [116], for B cells and follicular dendritic cells in explanted lymph nodes [117] and *in vivo* for immune cells exchanging lipids through nanotubes [118], in a process called trogocytosis. The acquisition of membrane dyes by non-labelled neighbouring cells has been shown to be particularly efficient, if the labelled cells are dead [115]. Optimization of the labelling protocol is critical for minimization of dye-induced toxicity, to reduce cell death and to decrease non-specific signals *in vivo*. For example, we optimized HSPC staining with DiD to 10 min of incubation at a final concentration of 0.5 µM, followed by immediate injection of the labelled cells in order to obtain the best cell viability as confirmed by functional testing [27,36]. Staining for longer periods of time or delaying injection of the labelled cells resulted in increased levels of DiD positive, non-autofluorescent shapes being detected in the bone marrow space during IVM sessions. A careful analysis of the shape and size of signals allowed discrimination of live, labelled cells from debris and autofluorescence. A control experiment based on double labelling of cells with a genetic reporter as well as the dye of interest provided the ideal way to determine the shape and size of signals that should be included in further analysis (figure 2). A similar control was described in a zebrafish study, in which human cells were double labelled with RFP- and human-specific antibodies [119].

### 4.2. Controlling for cell viability and function

**Figure 3.**
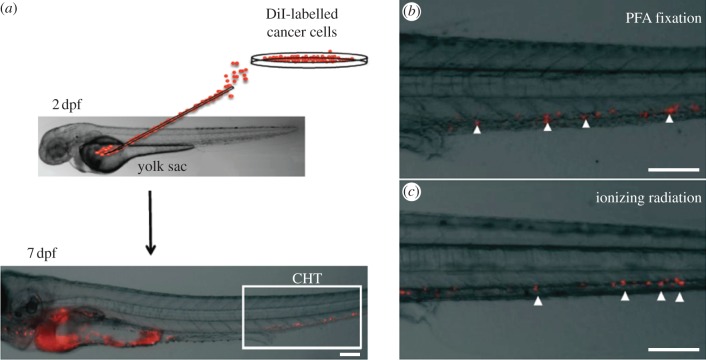
Live/dead control experimental design. (*a*) A549 cells labelled with CM-DiI were injected into the yolk sac of wild-type zebrafish embryos at 48 h post-fertilization and *in vivo* imaging was performed 5 days later. (*b*,*c*) Higher magnification images of the CHT area, where CM-DiI signal was detected despite cells being treated with toxic doses of PFA or ionizing radiation prior to injection. This level and type of CM-DiI signal therefore cannot be used to quantify micrometastasis formation. Scale bars, 100 µm.

Given that the highest amount of membrane dye transfer has been associated with cell death, it is critical to confirm the viability of imaged cells. Moreover, a thorough knowledge of the experimental model examined is necessary to draw the correct conclusions from imaging experiments. For example, it has been demonstrated that HSPCs injected into non-irradiated recipients do not engraft (i.e. they do not give rise to detectable progeny); however, they remain viable. When imaging HSPCs in non-irradiated recipients, we therefore identified signals of the expected shape and size for viable HSPCs; however, we did not draw conclusions about the physiological niches of these cells based on their location. Rather, we could do so for HSPCs observed in lethally irradiated recipients, where we were also able to test their long-term engraftment [36]. It is, however, not always possible to follow up the performance of the imaged cells: for example, human tumour cells injected into zebrafish embryos to study metastasis to various tissues do not all develop into lethal tumours and often are cleared to non-detectable levels. In this case, a useful control is the injection of non-viable cells in order to obtain information about levels of non-cell-associated staining. We performed such an experiment by treating A549 human lung adenocarcinoma cells with lethal doses of fixative or ionizing irradiation, labelling them with CM-DiI, injecting them in the yolk sac of 48 h old embryos and imaging them 5 days later (figure 3). At this time point, other groups working with other tumour cell lines have reported the presence of micrometastases in the caudal haematopoietic tissue (CHT), a region of active haematopoiesis during zebrafish embryonic development [108–112]. Interestingly, we also obtained signal in this area using dead cells, indicating that this particular experimental set-up does not provide a reliable measure of the metastatic potential of A549 cells.

### 4.3. Tissue macrophages as dye transfer acceptors

**Figure 4.**
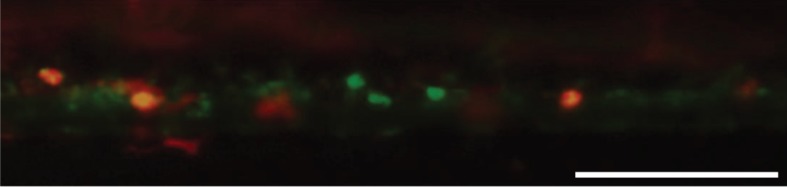
A549 cells labelled with DiO (green) were injected into the yolk sac of an *fms*:mCherry zebrafish at 48 h post-fertilization. Five days later partial overlap between DiO (A549 cells) and mCherry (macrophages) signals is detected (yellow), indicating that dye transfer has occurred, probably as a consequence of phagocytosis of dead A549 cells. Scale bar, 100 µm.

It is possible that fluorescent debris could accumulate within the tissue, but, in addition, tissue macrophages may actively acquire dye *in vivo* [120]. In fact, these cells have been labelled for *in vivo* imaging studies by intravenous injection of Texas red dextran [14], and a similar approach has been documented for labelling microglia in the retina [121] and dendritic cells in lymph nodes [122]. We injected live, DiO-labelled A549 cells into Tg(*fms*:mCherry) zebrafish embryos, in which macrophages express the mCherry fluorescent protein [50]. Five days following injection, as expected, DiO and mCherry double positive macrophages were detected in the CHT region (figure 4). DiO single positive signals could also result from DiO being taken up by other myeloid cells, debris accumulating in the extracellular space or residual viable A549 cells.

In general, unless tissue macrophages are visible through the expression of an endogenous reporter, it is impossible to evaluate the extent of dye transfer. For example, in the case of haematopoietic progenitor cells, it is obvious that irregular DiD signal is not cell associated; however, one cannot assume it corresponds to uptake by macrophages. In zebrafish, the shape of signal is not sufficient to identify viable labelled cells and further controls may be needed.

### 4.4. Best practice

In general, before making definitive conclusions on the suitability of lipophilic dyes as cell tracers for an experiment, it is important that the labelling protocol is optimized to minimize cell death by finding the appropriate balance between staining duration, washes and timing of injection. Moreover, cross-validation experiments should be performed to evaluate the impact of dye transfer, for example, by double labelling the injected cells. Finally, the viability of the injected cells must be assessed functionally by testing long-term engraftment of labelled cells, by performing live/dead control experiments or, ideally, by harvesting and functionally testing the labelled cells following imaging.

## 5. Recent and future developments

### 5.1. Near-infrared proteins and dyes

*In vivo* imaging of cell biology processes has been increasingly rewarding, thanks to the constant improvement of fluorophores, both proteins and chemical, and to parallel technological advances. Recent developments in these fields are already providing indications on the future avenues of IVM studies. For example, fluorescent proteins emitting in the near-infrared region (such as Neptune [53] and eqFP650/670 [54]) and the infrared region (such as iRFP [123,124]) are becoming popular because they are more efficiently excited and detected in living tissues, where light scattering, autofluorescence and absorption are highest at lower wavelengths [125,126]. These proteins have so far been successfully used for whole-body imaging experiments in mice [53,54,123,124] and more sensitive near-infrared photomultipliers (PMTs) built into confocal microscopes will make them promising candidates also for single-cell resolution *in vivo* imaging approaches. The same is true for near-infrared dyes: while several have been developed for whole-body imaging, few of them have been described in single-cell resolution *in vivo* imaging studies. One exception is DiR, which has been used for both single cell tracking in mouse bone marrow [101] and whole-body imaging of macrophages [127].

### 5.2. Fluorophores with large Stokes shift and quantum dots

While red fluorophores are currently imaged through confocal microscopy, optical parametric oscillator (OPO) technology is making red and far red fluorophores amenable to two-photon imaging [128], the ideal IVM modality as it reduces phototoxicity and increases contrast and resolution especially at higher tissue depths. Future generations of OPO technology could make even near-infrared fluorophores detectable with two-photon excitation. Alternatively, the development of fluorophores that are excitable by short wavelengths but emit far red and near-infrared signals provides an ideal labelling strategy for IVM. Keima, as well as LSS-mKate1 and LSS-mKate2, are proteins engineered to absorb similarly to CFP but emit above 600 nm, and other fluorescent proteins could be similarly engineered to allow two-photon excitation [129–131]. A simpler alternative to this complex engineering could be provided by the increasing availability of quantum dots. These small nanocrystals are made mainly of semiconductor materials such as cadmium, but also of carbon and silica and have unique optical properties compared with other conventional organic fluorophores. They have a high fluorescent yield, are highly photostable and couple narrow emission spectra with a wide range of absorption, allowing great flexibility in excitation, including UV excitation and far red/near-infrared emission [132]. It is therefore possible to use different types of quantum dots to label multiple cell types for simultaneous detection using just a single confocal or two-photon excitation wavelength, considerably reducing laser-induced photodamage of the tissue. Despite these superior optical properties and their successful application as vital labelling agents, for example in zebrafish [133] or to target cells *in vivo* [134,135], only a few studies have used quantum dots to label cells for transfer followed by *in vivo* imaging [65]. The limitations currently posed by quantum dot technology are the cost of the reagents, the efficiency of labelling protocols and their cytotoxicity, especially evident when quantum dots are introduced within the cytoplasm [136]. Rather than using them to label specific cell types, we found non-targeted quantum dots ideal to highlight the vascular network and we could easily select quantum dots whose excitation and emission spectra would not overlap with other fluorophores used simultaneously [36].

### 5.3. Antibodies and aptamers

A solution to efficient targeting of quantum dots and other fluorophores to specific cell types is provided by their conjugation to antibodies, an option currently gaining popularity for use in flow cytometry [53]. In fact, the use of fluorophore-conjugated antibodies is gaining popularity both for labelling cells *ex vivo* and especially for performing *in vivo* immunostaining. Through this approach, visualization of bone marrow vasculature areas rich in SDF1, selectins and VCAM1, lymphatic vessels and myeloid cells has been achieved [72,101,137,138]. Moreover, phycoerythrin-immune complexes were successfully used to specifically label follicular dendritic cells as well as macrophages in the splenic marginal zone [139,140]. *In vivo* immunolabelling is a very exciting development for IVM studies, as it avoids all *ex vivo* steps and, by bypassing transplantation of the cells of interest, allows observation of tissues under truly physiological conditions. The main challenges raised by the use of intact antibodies for *in vivo* labelling of cells and tissue structures are tissue penetration (i.e. the efficiency of labelling is likely to rapidly decrease with distance from vasculature), potential non-specific signal and aberrant immune cell responses. The last two problems, both due to the antibodies binding and activating cellular Fc receptors, can be alleviated by removal of the Fc part of the antibodies [141], or, alternatively, by working with fluorophore-conjugated domain antibodies, which contain only the variable portion of heavy and light chains. Domain antibodies are several times smaller than conventional antibodies and are therefore promising also in regard to efficient tissue penetration [142]. For example, fluorescent Fab fragments were used to *in vivo* label splenic lymphocytes, subsequently detected by IVM [141].

An alternative to antibodies may be provided by aptamers, short nucleotide sequences that can recognize specific protein domains. Because aptamers are small and change conformation upon binding of their target, they can be engineered so that specific binding releases a quencher or generates a FRET pair, thus reducing non-specific staining [143]. So far, aptamers have been used to label mesenchymal stem and progenitor cells *ex vivo* and to visualize them in mouse bone marrow following transplantation [143]. The small size of Fab fragments and aptamers is also potentially their main limitation: owing to limited availability of lysine residues for chromophore binding, they are often less bright than traditional antibodies. To overcome this problem, fluorescent dyes with bright signal and long lifetime fluorescence, such as Alexa and ATTO dyes, are promising candidates to be used [144,145].

### 5.4. Combining multiple labelling and label-free imaging strategies

As already proved by several studies, the combination of multiple types of labels spanning through the widest possible region of the electromagnetic spectrum provides the best results as it allows imaging of multiple cell types and tissue structures simultaneously. Visualization of the tissue vascular network provides useful topological landmarks as well as a better understanding of tissue organization. A wealth of ‘non-targeted’, low-reactive fluorescent dyes allow visualization of vasculature following intravenous injection. FITC and TRITC dextran [14,56], non-targeted quantum dots [36] and angiosense probes [146] are already available. Injection of fluorescent lectin allows visualization of vessel walls [147] and of fluorophore-conjugated antibodies allows specific vascular subdomains to be highlighted [72,101].

The detection of collagen through its second-harmonic generation signal provides further information about tissue organization and has proved useful in guiding imaging of lymph nodes and bone marrow, when combined with the use of fluorescent proteins, dyes and vascular labels [36,148]. In fact, detection of endogenous emission through two-photon excitation and fluorescence lifetime imaging is becoming a rapidly growing field within IVM research and is providing information about the metabolic state of tissues as well as further contrast signals [149,150]. Tissue contrast can be obtained also by means of expressing a fluorescent protein through viral transduction or direct DNA injection in the whole tissue or organ of interest [23,151–153]. In zebrafish, this has been obtained by injecting second-harmonic generation emitting nanocrystals in the cytoplasm of one-cell stage embryos [154].

Ratiometric imaging has been proved useful in discriminating structures labelled with fluorophores of partially overlapping spectra [79]. Recently developed spectral detectors, containing arrays of PMTs collecting signal across the full visible/near-infrared range, allow the full emission spectrum of each fluorophore used to be simultaneously collected. Coupled to mathematical decoding of the signal obtained (spectral unmixing), they bring the promise of greatly increasing the number of overlapping fluorophores that can be simultaneously imaged and resolved [155].

## 6. Conclusions

The field of *in vivo* imaging at single-cell resolution using confocal and two-photon microscopy is exciting, rapidly evolving and gaining increasing attention because it records biological events as they happen. It is important to constantly develop new experimental approaches and labelling methods to increase the range of biological questions that can be addressed, but it is critical to ensure that the observations performed reflect reality. To date, a steadily growing number of fluorescent proteins and dyes have been used to label cells of interest both endogenously and taking advantage of transplantation procedures. Control experiments allowing identification of the cells giving rise to the signal observed are fundamental to ensure the reliability of collected data. Moreover, the cyto- and phototoxicity of available dyes must be tested in order to select a labelling strategy that does not affect the viability of the cells. The intrinsic toxicity of dyes owing to intercalation within cellular structures or potential interference with signalling processes has to be taken into account. The ideal dye, completely non-toxic and entirely specific, will probably never exist and one will inevitably alter cells by labelling them. Further development of label-free imaging modalities, such as second-harmonic generation microscopy, Raman spectroscopy, reflectance and autofluorescence lifetime imaging will liberate us from the limitations of active cell-labelling strategies such as genetic or *ex vivo* manipulation; however, they are likely to bring their own set of technical challenges. No matter the breadth of the technological developments, we will have to accept that we are affecting living tissues by observing them. The strength of our observations resides in knowing how we may be affecting the tissues and taking that into account.
